# Retraction Note: Systematic review and meta-analysis of the epidemiology of Vancomycin-resistance *Staphylococcus aureus* isolates

**DOI:** 10.1186/s13756-023-01327-8

**Published:** 2023-11-03

**Authors:** Qianxing Wu, Niloofar Sabokroo, Yujie Wang, Marzieh Hashemian, Somayeh Karamollahi, Ebrahim Kouhsari

**Affiliations:** 1 The Medical Lab of Hainan Cancer Hospital, Haikou, 570,312 Hainan Province People’s Republic of China; 2 Department of Microbiology, School of Medicine, Shahid Sadoughi University of Medical Sciences, Yazd, Iran; 3 School of International Pharmaceutical Business, China Pharmaceutical University, Nanjing, 211198 Jiangsu Province People’s Republic of China; 4 Clinical Microbiology Research Center, Ilam University of Medical Sciences, Ilam, Iran; 5 Laboratory Sciences Research Center, Golestan University of Medical Sciences, Gorgan, Iran; 6 Department of Laboratory Sciences, Faculty of Paramedicine, Golestan University of Medical Sciences, Gorgan, Iran

**Retraction Note: Antimicrobial Resistance & Infection Control (2021) 10:101**

10.1186/s13756-021-00967-y

The Editor-in-Chief has retracted this article. An investigation by the Publisher found evidence to suggest that authorship for this article was offered for sale before the article was submitted to the journal. The authors did not state explicitly whether they agree to this retraction.

